# Eyeglasses and risk of COVID-19 transmission—analysis of the Virus Watch Community Cohort study

**DOI:** 10.1016/j.ijid.2023.10.021

**Published:** 2024-02

**Authors:** Annalan M.D. Navaratnam, Christopher O'Callaghan, Sarah Beale, Vincent Nguyen, Anna Aryee, Isobel Braithwaite, Thomas E. Byrne, Wing Lam Erica Fong, Ellen Fragaszy, Cyril Geismar, Susan Hoskins, Jana Kovar, Parth Patel, Madhumita Shrotri, Sophie Weber, Alexei Yavlinsky, Robert W. Aldridge, Andrew C. Hayward, Virus Watch Collaborative, Susan Michie, Pia Hardelid, Linda Wijlaars, Eleni Nastouli, Moira Spyer, Ben Killingley, Ingemar Cox, Vasileios Lampos, Rachel A. McKendry, Tao Cheng, Yunzhe Liu, Jo Gibbs, Richard Gilson, Alison Rogers, Anne M. Johnson

**Affiliations:** 1 Institute of Health Informatics, University College London, London, UK; 5 Institute for Global Health, University College London, London, UK; 6 Centre for Behaviour Change, University College London, London, UK; 7 Department of Population, Policy and Practice, UCL Great Ormond Street Institute of Child Health, London, UK; 8 Francis Crick Institute, London, UK; 9 Health Protection and Influenza Research Group, Division of Epidemiology and Public Health, University of Nottingham School of Medicine, Nottingham, UK; 10 University College London Hospital, London, UK; 11 Department of Computer Science, University College London, London, UK; 12 London Centre for Nanotechnology and Division of Medicine, University College London, London, UK; 13 SpaceTimeLab, Department of Civil, Environmental and Geomatic Engineering, University College London, London, UK; 1 Institute of Health Informatics, University College London, London, UK; 2 Infection, Immunity & Inflammation Department, Great Ormond Street Institute of Child Health, University College London, London, UK; 3 Institute of Epidemiology and Health Care, University College London, London, UK; 4 Department of Infectious Disease Epidemiology, London School of Hygiene and Tropical Medicine, London, UK

**Keywords:** Communicable disease, Infection control, Public health, Respiratory tract infections

## Abstract

**Objectives:**

The importance of SARS-CoV-2 transmission via the eyes is unknown, with previous studies mainly focusing on protective eyewear in healthcare settings. This study aimed to test the hypothesis that wearing eyeglasses is associated with a lower risk of COVID-19.

**Methods:**

Participants from the Virus Watch prospective community cohort study responded to a questionnaire on the use of eyeglasses and contact lenses. Infection was confirmed through data linkage, self-reported positive results, and, for a subgroup, monthly capillary antibody testing. Multivariable logistic regression models, controlling for age, sex, income, and occupation, were used to identify the odds of infection depending on frequency and purpose of eyeglasses or contact lenses use.

**Results:**

A total of 19,166 participants responded to the questionnaire, with 13,681 (71.3%, CI 70.7-72.0) reporting they wore eyeglasses. Multivariable logistic regression model showed a 15% lower odds of infection for those who reported using eyeglasses always for general use (odds ratio [OR] 0.85, 95% 0.77-0.95, *P* = 0.002) compared to those who never wore eyeglasses. The protective effect was reduced for those who said wearing eyeglasses interfered with mask-wearing and was absent for contact lens wearers.

**Conclusions:**

People who wear eyeglasses have a moderate reduction in risk of COVID-19 infection, highlighting that eye protection may make a valuable contribution to the reduction of transmission in community and healthcare settings.

## Highlights

• Eyeglasses are associated with a protective effect against COVID-19.
• Protective effect was reduced if wearing glasses interfered with mask-wearing.
• There was no protective effect for those who wore contact lenses.
• There was still a protective association after adjusting for age, sex, occupation, and income.

## Introduction

Respiratory viruses infect individuals via the nose, mouth, and eyes, through contact with surfaces touched by the individual, or via small and larger (i.e., droplet) aerosol particles [1]. Recommendations for the protection of the general public in most countries include social distancing, handwashing, and face mask use but not eye protection. In the UK, eye protection (including full-face visors or goggles) is recommended in healthcare settings if blood or body fluid contamination to the eyes or face is anticipated or likely. In addition, when caring for patients with a suspected or confirmed infection spread by the droplet or airborne route as deemed necessary by a risk assessment, or during aerosol-generating procedures [2]. Regular corrective eyeglasses are not considered eye protection.

The eyes present two routes for SARS-CoV-2 infection, the first through infection of conjunctival cells that contain angiotensin-converting enzyme 2 receptors. Several studies have detected SARS-CoV-2 ribonucleic acid in the tear film, conjunctiva, and conjunctival sac with between 1-12% of patients with COVID-19 reported to have ocular manifestations [3], [4], [5], [6]. The second infection route is via the nasolacrimal duct, which is known to transport pathogens to the nose within minutes and onward to the nasopharynx [7]. Supporting the eye as a route of SARS-CoV-2 infection, conjunctival inoculation of the virus in macaques leads to interstitial pneumonia [8]. A small number of hospital-based observational studies suggest that eye protection may help prevent COVID-19 [9]. The earliest of which was an observational study of 276 patients with COVID-19 admitted to a hospital and found the proportion of spectacle wearers was lower than the general population [10].

Based on the biological mechanisms and studies in healthcare we hypothesized that eyeglasses wearing in community settings would reduce the risk of COVID-19. Eyeglasses may provide a barrier to prevent exposure to infectious aerosol particles, particularly the ballistic component of larger particles, and may also reduce contaminated fingers touching the eyes. We do not expect to see this same protective effect in a counterfactual contact lens analysis. We therefore developed a survey on eyeglasses and contact lenses within the Virus Watch cohort to test these hypotheses. The aim of this study was to test the hypothesis that wearing eyeglasses is associated with a lower risk of COVID-19.

## Methods

The Virus Watch study is a household community cohort of acute respiratory infections in England and Wales that started recruitment in June 2020 [11]. As of 2 February 2022, 58,670 participants were recruited using a range of methods (e.g. post, social media, General Practice letters) and participants provided information on registration including age, sex, occupation, and household information (e.g. household income). In the December 2021 monthly questionnaire, 31,749 participants were asked whether they used eyeglasses or contact lenses, and if so, how frequently they used them generally, for reading, or for carrying out a specific task. They were also asked about the level of agreement with the following statement: ‘I am less likely to wear a face covering when I have my glasses on because my glasses steam up’.

The covariates considered in this analysis were age, sex, income, and occupation. Income was defined as the combined household income divided by the number of adults in that household. This was then put into categories ranging from £0-9,999 to £80,000+, with intervals of £10,000. Self-reported occupation was grouped using the Office for National Statistics' Standard Occupational Classification Hierarchy [12]. ‘Skilled trades occupations’ and ‘Process, plant and machine operatives’ were grouped into ‘manual’, with all other occupations grouped as ‘non-manual’. If occupation was not available, ‘not reported’ was recorded.

There were multiple ways in which to identify the first SARS-CoV-2 infection among participants of this study. Infection was identified based on the first positive result from the following sources:

1. Data are linked to the Second-Generation Surveillance System (SGSS), which contains SARS-CoV-2 test results using data from hospitalizations (Pillar 1) and community testing (Pillar 2). Linkage was conducted by National Health Service Digital with the linkage variables being sent in March 2021. The linkage period for SGSS Pillar 1 encompassed data from March 2020 until August 2021 and from June 2020 until November 2021 for Pillar 2.
2. Self-reported positive polymerase chain reaction (PCR) or lateral flow device swabs for SARS-CoV-2 infection as part of the Virus Watch weekly survey.
3. Monthly self-collected capillary blood samples (400-600µL) in a subsample of 11,701 participants, which were tested in United Kingdom Accreditation Service (UKAS)-accredited laboratories. Serological testing using Roche's Elecsys Anti-SARS-CoV-2 electrochemiluminescence assays targeting total immunoglobulin (Ig) to the Nucleocapsid (N) protein, or to the receptor binding domain in the S1 subunit of the Spike protein (S) (Roche Diagnostics, Basel, Switzerland). At the manufacturer-recommended seropositivity thresholds (≥1.0 cut-off index for N and ≥0.8 units per milliliter [U/ml] for S) a positive result was defined based on positivity to the N protein.
4. Clinical-collected venous blood samples tested for the S protein. In-clinic serology was conducted twice per participant between September 2020-January 2021 (Autumn round n = 3050) and April 2021-July 2021 (Spring round n = 2775)) (see study protocol for details) [12]. Positivity was defined as evidence of S-positivity in absence of receiving any COVID-19 vaccination before the serological test.

We used sliding date window matching (14-day window) to identify positive tests recorded by both Virus Watch and linkage to UK national records; where both were available, the linkage date was used. Where both swab and serological positives were recorded, we used the PCR/LFT date, unless the serological positive occurred first. Reinfections were not included.

### Outcomes

The primary outcome is the risk of infection depending on self-reported use of eyeglasses, grouped into frequency of use. Frequency of use in the questionnaire was reported as ‘Never’, ‘Rarely’, ‘Sometimes’, ‘Most times’, and ‘Always’. ‘Rarely’, ‘Sometimes’, and ‘Most times’ were then grouped into ‘Sometimes’.

Secondary outcomes were risk of infection depending on the use of mask and eyeglasses at the same time as well as the frequency of use of contact lenses (for counterfactual analysis).

### Analysis

All respondents to the December 2021 survey were included in the analysis. It was assumed that if participants did not have a positive test, they did not have previous SARS-CoV-2 infection. The first date of infection was used and subsequent infections were excluded. Proportions of positive individuals were calculated with 95% CIs. Multivariable logistic regression models included age (as a continuous variable), sex, household income per adult in the household, and occupation. For the comparison of binary variables, chi-square test was used. All analyses were carried out with R-studio (R 4.0.5.) using packages; ‘tidyverse’, ‘ggplot2’, and ‘rstatix’.

## Results

Of 31,749 invited to answer the monthly survey on eyeglasses and contact lens use, there were 19,166 respondents. The median age was 63 years old (IQR 52-70) and 10,470 participants were female (54.6%, 95% CI 53.9-55.3%). A total of 13,681 participants (71.3%, CI 70.7-72.0) reported wearing eyeglasses. 19.6% (3,757, 95% CI 19.0-20.2) had evidence of previous COVID-19 infection. There was also no difference between sex, with 19.6% (8,255, 95% CI 18.7-20.5) of males and 19.9% females (10,470, 95% CI 19.1-20.6) having evidence of a previous infection.

**Figure 1:**
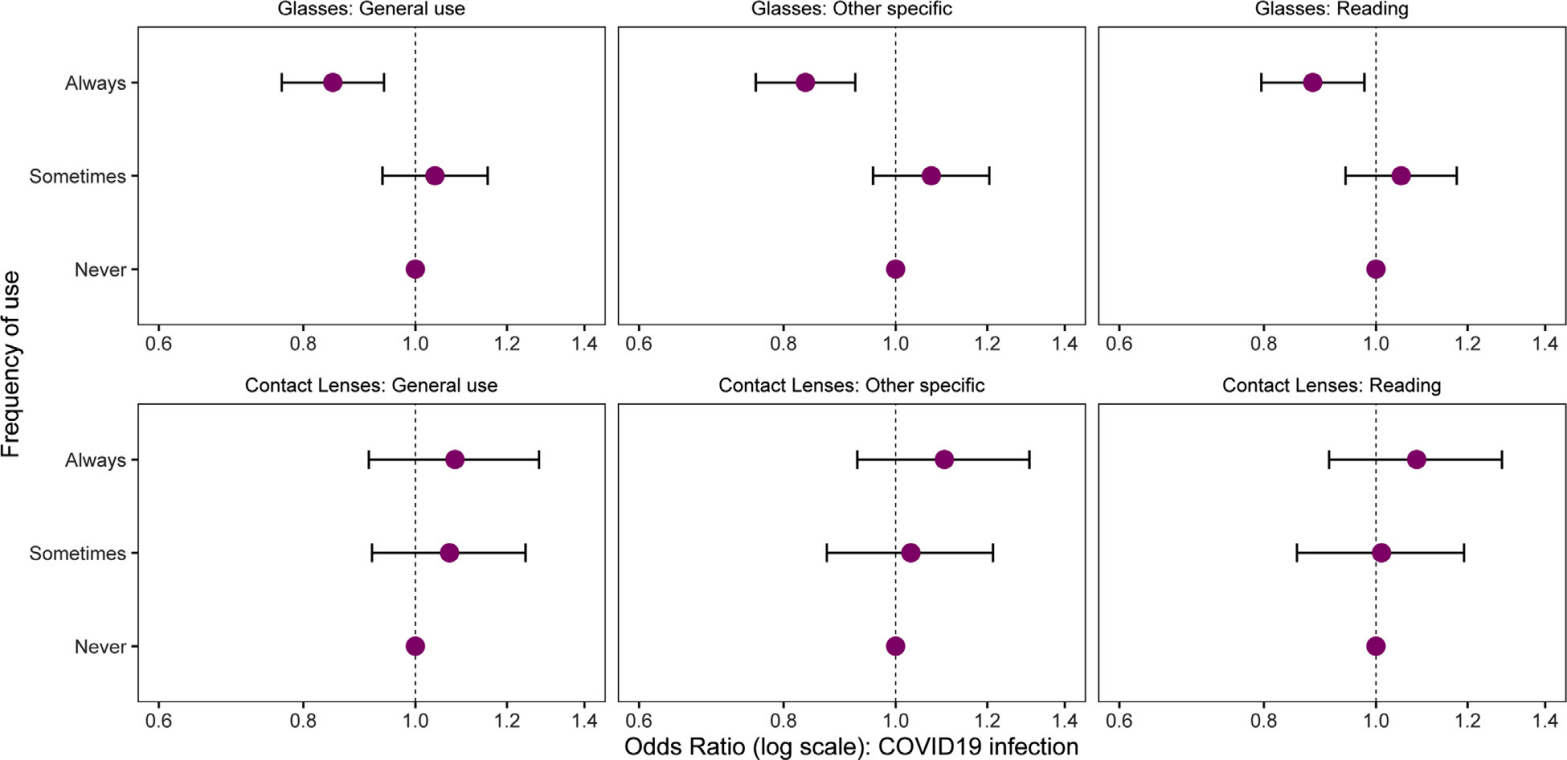
Adjusted odds ratios and 95% confidence intervals showing association of COVID-19 infection with glasses and contact lenses use. Adjusted for age, sex, income and occupation.

Among those who never wore eyeglasses for general use, 22.99% (95% CI 22.01-23.97%) were infected versus 15.63% (95% CI 14.76-16.5%; OR 0.62 95% CI 0.57-0.68) for those who always wore eyeglasses for general use (Table 1). Multivariable logistic regression model, adjusting for age, sex, income, and occupation, showed 15% lower odds of infection for those who reported always using eyeglasses for general use (OR 0.85, 95% 0.77-0.94, *P* = 0.002) compared to non-wearers (Figure 1). This was similar to always using eyeglasses for reading and other specific tasks, but not for any frequency or purpose of using contact lenses (Figure 1).

**Table 1:** Summary of the proportion of individuals with previous SARS-CoV-2 infection grouped by type and frequency of use with 95% CIs. Missing refers to missing data.

Eyewear	Usage	Frequency	Total Responses	Positives n (%)	95% CI
Glasses	General use	Never	7047	1620 (22.99)	22.01, 23.97
		Sometimes	4959	1002 (20.21)	19.09, 21.32
		Always	6687	1045 (15.63)	14.76, 16.5
		Missing	473	90 (19.03)	15.49, 22.56
	Other specific	Never	7077	1640 (23.17)	22.19, 24.16
		Sometimes	3383	711 (21.02)	19.64, 22.39
		Always	7405	1172 (15.83)	15, 16.66
		Missing	1301	234 (17.99)	15.9, 20.07
	Reading	Never	5934	1401 (23.61)	22.53, 24.69
		Sometimes	4695	946 (20.15)	19, 21.3
		Always	7948	1299 (16.34)	15.53, 17.16
		Missing	589	111 (18.85)	15.69, 22
Contact Lenses	General use	Never	15718	3074 (19.56)	18.94, 20.18
		Sometimes	1333	303 (22.73)	20.48, 24.98
		Always	1131	222 (19.63)	17.31, 21.94
		Missing	984	158 (16.06)	13.76, 18.35
	Other specific	Never	15291	3007 (19.67)	19.04, 20.3
		Sometimes	1162	257 (22.12)	19.73, 24.5
		Always	1086	215 (19.8)	17.43, 22.17
		Missing	1627	278 (17.09)	15.26, 18.92
	Reading	Never	15422	3036 (19.69)	19.06, 20.31
		Sometimes	1168	256 (21.92)	19.55, 24.29
		Always	1096	214 (19.53)	17.18, 21.87
		Missing	1480	251 (16.96)	15.05, 18.87

**Figure 2:**
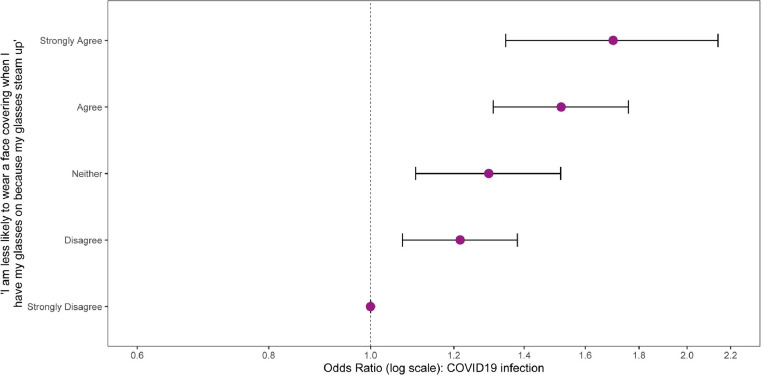
Adjusted odds ratios and 95% Confidence intervals of COVID-19 infection, comparing glasses users according to their agreement with the question “I am less likely to wear a face covering when I have my glasses on because my glasses steam up”. Adjusted for age, sex, income and occupation.

When eyeglasses wearers were asked if they agreed with the statement ‘I am less likely to wear a face covering when I have my glasses on because my glasses steam up’ the proportion of positives was lowest for ‘Strongly Disagree’ (15.31%, 95% CI 14.42;16.2) and highest for ‘Strongly Agree’ (25.09%, 95% CI 21.42;28.77). This linear association remained after adjusting for age, sex, income, and occupation, suggesting that when eyeglasses interfered with mask use there was a reduction in protective effect (Table 2 and Figure 2).

**Table 2:** Summary of the proportion of individuals with previous SARS-CoV-2 infection grouped by level of agreement with ‘I am less likely to wear a face covering when I have my glasses on because my glasses steam up’’ with 95% CIs.

Response	Total Responses	Positives (%)	95% CI
Strongly Disagree	6257	958 (15.31)	14.42, 16.2
Disagree	3336	608 (18.23)	16.92, 19.54
Neither	1574	311 (19.76)	17.79, 21.73
Agree	1774	387 (21.82)	19.89, 23.74
Strongly Agree	534	134 (25.09)	21.42, 28.77
Missing data	5691	1359 (23.88)	22.77, 24.99

## Discussion

Our results show a significant reduction in the odds of COVID-19 infection among those who always wear eyeglasses. This demonstrates the importance of the eyes as a portal for infection and suggests that strategies to broaden the use of eye protection could help prevent transmission and contribute to infection control. The counterfactual analysis of contact lenses showed no protective effect, strongly suggesting a causal relationship between eyeglasses wearing and reduced risk of SARS-CoV-2 infection. The findings also highlight that many individuals reduce mask-wearing with eyeglasses, because of eyeglasses steaming up, which is associated with a reduced protective effect. This suggests the need for mask design and usage to prevent steaming up during use as well as adds to growing evidence of face coverings’ protective effect.

Other observational studies outside of the hospital setting have reported different outcomes. Lehrer et al. [13] linked records from the UK biobank to test results of the National Health Service's COVID-19 laboratories, which were largely limited to hospital inpatients at the time. They reported a lower risk of infection for those wearing eyeglasses, adjusting for age and sex, but were unable to separate contact lens users from eyeglasses users. It was also unclear if the control group had negative test results or were just not tested. A cohort study of 1,279 and 841 rescue and emergency service employees in Denmark and Sweden, respectively, found inconclusive evidence when adjusting for age, sex, job function, and number of workday contacts [14]. Direction of effect sizes differed between countries, that is, Sweden showed a protective association, which may reflect differences in seroprevalence and general country-specific preventive measures. Furthermore, it is likely they had insufficient power to detect a difference after adjusting for all included covariates. The findings from a community-based randomized control trial of 3,717 participants in Norway were also inconclusive, but participants had reduced access to testing due to national policy changes and the analysis only accounted for age and sex [15].

There are a number of plausible mechanisms by which wearing eyeglasses could contribute to the prevention of COVID-19 infection. Healthy individuals involuntarily touch their eyes around three times per hour and wearing spectacles may reduce the number of times SARS-CoV-2 contaminated fingers touch the eyes [16]. It is likely that spectacles present a barrier to the direct impaction of viruses on the eyes. Eye deposition of SARS-CoV-2 may also occur directly from the impaction of the ballistic component of aerosol particles, particularly larger particles (i.e. droplets) produced by coughing and sneezing. Air currents may also direct virus-containing aerosolized particles toward the eye, enhancing deposition, and Brownian motion of aerosolized particles may also result in deposition on the ocular surface.

We hypothesize that even greater protection against COVID-19 may be afforded by eye protection that wraps around the eyes or seals the eyes from the environment. Face shields are frequently used in hospitals and by the public in some countries. Like eyeglasses, they may reduce infection risk to the ballistic component of droplets, but they do not offer full protection against small aerosol particles, as illustrated by experimental studies [17]. Protection afforded by eye protection is also likely to be seen for other respiratory viruses, such as influenza and respiratory syncytial virus, which remain infectious in exhaled aerosol particles [18,19].

The counterfactual absence of a protective effect in contact lens wearers also helps to strengthen the findings. The absence of protective effect with contact lenses aligns with the biological mechanisms of SARS-CoV-2 infection through the eyes. Hands are a vector of transmission, and using contact lenses is associated with increased contamination from fingertips due to application, removal, and adjustment (because of dry eyes and irritation) of lenses [20], [21], [22], [23]. Furthermore, as contact lenses only cover the cornea of the eye, there is no protection from the two routes of infection, the conjunctiva and nasolacrimal ducts. Unadjusted and adjusted odds ratios for infection differed substantially, which may be explained by the strong influence of age on eyeglasses use. Younger people are more likely to be nearsighted and therefore eyeglasses for myopia are more likely to be used in social situations. Whereas older people are prone to be farsighted, which is less likely to require use of eyeglasses in social situations [24].

Strengths of this work include the prospective approach, large number of participants, multiple approaches to capture SARS-CoV-2 infections, and adjustment for a wide range of potential confounders [9,[13], [14], [15]. We considered the need for visual correction is strongly influenced by age but not by other variables that may impact on risk of infection. However, choice of contact lenses versus eyeglasses may be affected by social factors and occupation and poor visual acuity may prevent people from working in some occupations. It was, therefore, important to be able to control these variables. As our questions are specific to eyeglasses, not inclusive of face shields, we have overcome concerns about whether the reduced transmission is through reduced inhalation or protecting eyes [9]. An assumption in our analysis however is that the reported frequency of use was reflective of the entire study period. More specific and detailed questions on the context of eyeglass wearing (e.g. social or commuter settings) have been commented as a limitation of this analysis [22]. As these questions were part of the monthly routine survey questionnaire, in addition to weekly questionnaires, a compromise needed to be reached between depth of information and survey attrition. We also note that the benefit of glasses wearing may be greater in those with the most exposure (e.g., those unable to work from home or working in healthcare facilities), but we did not have sufficient power to explore these hypotheses.

Although community-based, the findings of this study show the potential of eye protection to reduce infection risk and may be particularly important in high-exposure settings such as healthcare. Eye protection has been reported to be the most frequently missed item of personal protective equipment among healthcare workers, emphasizing the importance of providing evidence of its benefits [25]. Our work adds to existing observational studies extending the evidence of the protective effects beyond healthcare workers and into community settings.

## Conclusion

Extending the use of protective eyewear should be considered as part of broader strategies to prevent community transmission of infection and may be valuable to consider in the event of future pandemics and in high-exposure occupations including healthcare.

## Declaration of competing interests

ACH serves on the UK New and Emerging Respiratory Virus Threats Advisory Group. AMJ was a Governor of Wellcome Trust from 2011-2018 and is Chair of the Committee for Strategic Coordination for Health of the Public Research.

## References

[1] Belser JA, Rota PA, Tumpey TM (2013). Ocular tropism of respiratory viruses. Microbiol Mol Biol Rev.

[2] GOVUK. Infection prevention and control for seasonal respiratory infections in health and care settings (including SARS-CoV-2) for winter 2021 to 2022, https://www.gov.uk/government/publications/wuhan-novel-coronavirus-infection-prevention-and-control/covid-19-guidance-for-maintaining-services-within-health-and-care-settings-infection-prevention-and-control-recommendations; n.d. [accessed 20 March 2022].

[3] Siedlecki J, Brantl V, Schworm B, Mayer WJ, Gerhardt M, Michalakis S (2020). COVID-19: ophthalmological aspects of the SARS-CoV 2 global pandemic. Klin Monbl Augenheilkd.

[4] Xia J, Tong J, Liu M, Shen Y, Guo D (2020). Evaluation of coronavirus in tears and conjunctival secretions of patients with SARS-CoV-2 infection. J Med Virol.

[5] Seah I, Agrawal R (2020). Can the coronavirus disease 2019 (COVID-19) affect the eyes? A review of coronaviruses and ocular implications in humans and animals. Ocul Immunol Inflamm.

[6] Zhang X, Chen X, Chen L, Deng C, Zou X, Liu W (2020). The evidence of SARS-CoV-2 infection on ocular surface. Ocul Surf.

[7] Maxcy KF (1919). The transmission of infection through the eye. JAMA.

[8] Deng C, Yang Y, Chen H, Chen W, Chen Z, Ma K (2020). Low risk of SARS-CoV-2 transmission through the ocular surface. Acta Ophthalmol.

[9] Byambasuren O, Beller E, Clark J, Collignon P, Glasziou P (2021). The effect of eye protection on SARS-CoV-2 transmission: a systematic review. Antimicrob Resist Infect Control.

[10] Zeng W, Wang X, Li J, Yang Y, Qiu X, Song P (2020). Association of daily wear of eyeglasses with susceptibility to coronavirus disease 2019 infection. JAMA Ophthalmol.

[11] Hayward A, Fragaszy E, Kovar J, Nguyen V, Beale S, Byrne T (2021). Risk factors, symptom reporting, healthcare-seeking behaviour and adherence to public health guidance: protocol for Virus Watch, a prospective community cohort study. BMJ Open.

[12] Beale S, Hoskins S, Byrne T, Fong E, Fragaszy E, Geismar C (2023). Differential risk of SARS-CoV-2 infection by occupation: evidence from the Virus Watch prospective cohort study in England and Wales. J Occup Med Toxicol.

[13] Lehrer S, Rheinstein P (2021). Eyeglasses reduce risk of COVID-19 infection. In Vivo.

[14] Gregersen R, Jacobsen RK, Laursen J, Mobech R, Ostrowski SR, Iversen K (2022). Association of COVID-19 infection with wearing glasses in a high-prevalence area in Denmark and Sweden. JAMA Ophthalmol.

[15] Fretheim A, Elgersma IH, Helleve A, Elstrøm P, Kacelnik O, Hemkens LG (2022). Effect of wearing glasses on risk of infection with SARS-CoV-2 in the community: a randomized clinical trial. JAMA Netw Open.

[16] Kwok YLA, Gralton J, McLaws ML (2015). Face touching: A frequent habit that has implications for hand hygiene. Am J Infect Control.

[17] Lindsley WG, Noti JD, Blachere FM, Szalajda JV, Beezhold DH (2014). Efficacy of face shields against cough aerosol droplets from a cough simulator. J Occup Environ Hyg.

[18] Leung NHL (2021). Transmissibility and transmission of respiratory viruses. Nat Rev Microbiol.

[19] Kulkarni H, Smith CM, Lee DDH, Hirst RA, Easton AJ, O'Callaghan C (2016). Evidence of respiratory syncytial virus spread by aerosol. Time to Revisit Infection Control Strategies?. Am J Respir Crit Care Med.

[20] American Academy Ophthalmology. Eye care during COVID-19: masks, vaccines and procedures, https://www.aao.org/eye-health/tips-prevention/coronavirus-covid19-eye-infection-pinkeye; 2021 [accessed 17 March 2022].

[21] Fonn D, Jones L (2019). Hand hygiene is linked to microbial keratitis and corneal inflammatory events. Cont Lens Anterior Eye.

[22] Markoulli M, Kolanu S (2017). Contact lens wear and dry eyes: challenges and solutions. Clin Optom (Auckl).

[23] Bhargava R (2020). Contact lens use at the time of SARS-CoV-2 pandemic for healthcare workers. Indian J Med Res.

[24] Guo Q, Xu J, Wei Y (2023). Comment on the virus watch community cohort study about glasses and risk of COVID-19 transmission. Asian J Surg.

[25] Burke RM, Balter S, Barnes E, Barry V, Bartlett K, Beer KD (2020). Enhanced contact investigations for nine early travel-related cases of SARS-CoV-2 in the United States. PLoS One.

